# Association of neighborhood-level sociodemographic factors with Direct-to-Consumer (DTC) distribution of COVID-19 rapid antigen tests in 5 US communities

**DOI:** 10.1186/s12889-023-16642-3

**Published:** 2023-09-22

**Authors:** Carly Herbert, Qiming Shi, Jonggyu Baek, Biqi Wang, Vik Kheterpal, Christopher Nowak, Thejas Suvarna, Aditi Singh, Paul Hartin, Basyl Durnam, Summer Schrader, Emma Harman, Ben Gerber, Bruce Barton, Adrian Zai, Michael Cohen-Wolkowiez, Giselle Corbie-Smith, Warren Kibbe, Juan Marquez, Nathaniel Hafer, John Broach, Honghuang Lin, William Heetderks, David D McManus, Apurv Soni

**Affiliations:** 1 Program in Digital Medicine, Department of Medicine, University of Massachusetts Chan Medical School, 55 Lake Avenue North, WorcesterWorcester, MA 01655 USA; 2 Center for Clinical and Translational Science, University of Massachusetts, University of Massachusetts Chan Medical School, Worcester, MA USA; 3 Department of Population and Quantitative Health Sciences, University of Massachusetts Chan Medical School, Worcester, MA USA; 4 CareEvolution LLC, Ann Arbor, MI USA; 5 Department of Pediatrics, Duke University School of Medicine, Durham, NC USA; 6 Department of Social Medicine, Department of Medicine, Center for Health Equity Research, University of North Carolina School of Medicine, Chapel Hill, NC USA; 7 Department of Biostatistics and Bioinformatics, Duke University School of Medicine, Durham, NC USA; 8 Washtenaw County Health Department, Washtenaw, MI USA; 9 Department of Emergency Medicine, University of Massachusetts Chan Medical School, Worcester, MA USA; 10 National Institute of Biomedical Imaging and Bioengineering, NIH, Via Contract With Kelly Services, Bethesda, MD USA; 11 Division of Cardiology, Department of Medicine, University of Massachusetts Chan Medical School, Worcester, MA USA; 12 Division of Health System Science, Department of Medicine, University of Massachusetts Chan Medical School, Worcester, MA USA

**Keywords:** COVID-19, Rapid antigen tests, Geospatial analysis, Direct-to-consumer

## Abstract

**Background:**

Many interventions for widescale distribution of rapid antigen tests for COVID-19 have utilized online, direct-to-consumer (DTC) ordering systems; however, little is known about the sociodemographic characteristics of home-test users. We aimed to characterize the patterns of online orders for rapid antigen tests and determine geospatial and temporal associations with neighborhood characteristics and community incidence of COVID-19, respectively.

**Methods:**

This observational study analyzed online, DTC orders for rapid antigen test kits from beneficiaries of the Say Yes! Covid Test program from March to November 2021 in five communities: Louisville, Kentucky; Indianapolis, Indiana; Fulton County, Georgia; O’ahu, Hawaii; and Ann Arbor/Ypsilanti, Michigan. Using spatial autoregressive models, we assessed the geospatial associations of test kit distribution with Census block-level education, income, age, population density, and racial distribution and Census tract-level Social Vulnerability Index. Lag association analyses were used to measure the association between online rapid antigen kit orders and community-level COVID-19 incidence.

**Results:**

In total, 164,402 DTC test kits were ordered during the intervention. Distribution of tests at all sites were significantly geospatially clustered at the block-group level (Moran’s I: *p *< 0.001); however, education, income, age, population density, race, and social vulnerability index were inconsistently associated with test orders across sites. In Michigan, Georgia, and Kentucky, there were strong associations between same-day COVID-19 incidence and test kit orders (Michigan: *r* = 0.89, Georgia: *r* = 0.85, Kentucky: *r* = 0.75). The incidence of COVID-19 during the current day and the previous 6-days increased current DTC orders by 9.0 (95% CI = 1.7, 16.3), 3.0 (95% CI = 1.3, 4.6), and 6.8 (95% CI = 3.4, 10.2) in Michigan, Georgia, and Kentucky, respectively. There was no same-day or 6-day lagged correlation between test kit orders and COVID-19 incidence in Indiana.

**Conclusions:**

Our findings suggest that online ordering is not associated with geospatial clustering based on sociodemographic characteristics. Observed temporal preferences for DTC ordering can guide public health messaging around DTC testing programs.

**Supplementary Information:**

The online version contains supplementary material available at 10.1186/s12889-023-16642-3.

## Introduction

Widespread, accessible testing is important to detect infection and reduce the risk of transmission for COVID-19 [1, 2]. However, throughout the COVID-19 pandemic, inequities in access and utilization of COVID-19 tests have been reported, and social vulnerability (e.g., racial and ethnic minorities, low income) has been associated with decreased geographic access to testing, reinforcing structural inequalities [3–7]. Rapid antigen tests (Ag-RDT) for COVID-19 may expand testing access throughout the United States due to their accessibility and ability to be used outside traditional healthcare settings.

With the shift to home-based testing, little is known about the sociodemographic characteristics of home-test users and if the distribution of Ag-RDT mimics preexisting disparities in access to COVID-19 testing. Generally, Ag-RDT users are likely to exhibit other COVID-19 protective behaviors, including mask use and social distancing, which are behaviors less common among rural and lower-income populations, as well as those with less education [8–10]. Therefore, it is important to understand the distribution of rapid home-testing to understand the current landscape of COVID-19 testing and promote health equity.

Further, little is known about factors that influence the uptake of home-tests, including the relationship between COVID-19 incidence or seasonality and home-testing usage. Previous studies have shown that incentivization and other cost-saving measures increase uptake of home-tests; however, it is unclear how individuals’ testing behavior reflect their personal risk of COVID-19 and lifestyle [11, 12].

Say Yes! Covid Test (SYCT!) was launched in the spring of 2021 by the Centers for Disease Control and Prevention (CDC) and National Institutes of Health (NIH) with the goal of distributing large numbers of free Ag-RDT to communities across the United States with a high burden of SARS-CoV-2 during 2021 [13]. This study aims to describe the sociodemographic, geographic, and temporal distribution of digitally ordered Ag-RDT kits and ordering patterns with respect to changing local COVID-19 prevalence and seasonality. We hypothesized that racial and socioeconomic disparities in access to testing would be present among the recipients of home-test kits and increased community prevalence of COVID-19 would be correlated with more home-test kit orders.

## Methods

### SYCT! Intervention communities and procedures

SYCT! intervention communities were chosen strategically by the NIH and CDC to target communities with lower vaccination rates and lower socioeconomic status. Five communities that received the intervention between June and November 2021 were included in this analysis: Ann Arbor/Ypsilanti, Michigan; Fulton County, Georgia; O’Ahu, Hawaii; Louisville, Kentucky; Indianapolis, IN. The interventions were restricted to residents of the indicated cities/counties. Free test kits were distributed either via online ordering and direct shipment to resident’s homes (direct-to-consumer, DTC) or local pick-up sites such as churches, schools, and community events. Each household was only allowed to order 1 test kit. More details about the SYCT intervention can be found elsewhere [12, 14–16]. The University of Massachusetts Institutional Review Board reviewed and determined this study was exempt because there was no collection or use of personal identifiable information.

### Digital assistant data collection

All DTC orders were processed through an online platform, developed by CareEvolution, and number of DTC orders per day for all eligible zip codes were recorded at the 9-digit zip-code level. All orders were deidentified using anonymous participant identifiers.

### Geospatial analyses

Number of DTC orders were tabulated by 9-digit zip code, and zip codes were converted to census block and geocoded to generate the number of test kit orders per census block group. Geographic shapefiles for 2018 Census block groups were obtained through the Census website (https://www.census.gov/cgi-bin/geo/shapefiles/index.php). To examine whether any spatial correlation existed in the number of DTC orders, we used Moran’s I statistics based on the Queen’s definition, meaning neighbors are defined when two areas share any border [17, 18]. A contiguity or adjacency weighting matrix was constructed.

Demographic data were obtained from the 2018 American Community Survey (ACS) and matched to appropriate 9-digit zip code regions using Census block identifiers, and the 2018 CDC Social Vulnerability Index (SVI) was matched at the census tract levels. Social Vulnerability Index is a measure created by the CDC to quantify and compare the social vulnerability of communities. The index includes 16 social factors derived from ACS data which are grouped into four themes: socioeconomic status (percent of population below poverty level, unemployed, without a high school diploma, and median household income), household characteristics (percent of population aged 65 and older, aged 17 or younger, with a disability, and single-parent households), racial and ethnic minority status (percent of population that are minorities (i.e., race other than non-Hispanic White) and speaks English “less than well”), and housing type/transportation (multi-unit strucures, mobile homes, crowding, no vehicle, and group quarters). Census tracts are compared and ranked against other tracts at the state-wide level based on percentiles, with 0 indicating low vulnerability and 1 indicating the highest vulnerability. Additional information about the calculation of the CDC SVI measure and ACS tables used to formulate these variables can be found elsewhere [19].

To determine whether spatial autocorrelation of DTC orders was explained by demographic factors, we fitted unadjusted and adjusted spatially autoregressive models [20, 21]. Specifically, we used a spatial Durbin model of the following form: $Y=\rho WY+X\beta +\epsilon$, where $W$ is the spatial weight matrix based on the Queen’s definition; the parameter $\rho$ is the spatial correlation; $X$ is a set of covariates with corresponding regression coefficients $\beta$; the residual error $\epsilon$ assumed to follow a normal distribution. Unadjusted model included only the intercept in $X$. The adjusted model included variables at the census block group and census tract levels to characterize the recipients’ neighborhoods. The model included race (percent of population that is Black, White, and Asian), population density, median income, median age, and percent of adults with a Bachelor’s degree at the block group level. The model also included the four SVI theme variables (socioeconomic status, household characteristics, racial and ethnic minority status, and housing type/transportation) at the census tract level. As the SVI measures and demographic variables were at different geographic levels, we were not concerned about co-linearity, as the block group and census tract measures added unique details about the community landscape. Analyses were conducted using the “spdep” package in R [22].

### Temporal association analyses

Temporal association analyses were conducted starting after the initial spike of DTC orders, based on visual determination, through the end of the distribution period. Hawaii was excluded from lag association analyses, as 94.5% of DTC orders occurred within a seven-day window, limiting the ability to analyze DTC orders over time. The Johns Hopkins COVID19 Tracker was used to abstract seven-day average COVID-19 incidence in each intervention community for the duration of the distribution period [23]. Seven-day average COVID-19 incidence, as opposed to daily incidence, was used to account for daily fluctuations in local public health reporting. While we hypothesized that testing behaviors may be influenced by COVID-19 incidence, there is a lag time in COVID-19 reporting which may influence when people adopt protective measures [24]. Therefore, to explore any potential temporal lagged association between the number of DTC orders ($Y$) and the number of 7-day average positive cases ($X$), we created lagged covariates; ${X}_{l}$, $l=0, 1, 2, 3, \dots , L$, for the $l$-day lagged (delayed) association between the number of positive cases and number of DTC orders. The maximum lag $L$ was determined as a half window length from the data analysis window. We then implemented a distributed lag model to estimate the lagged association between number of orders and number of positive cases [25–27]. Specifically, we used the following model: ${E[Y}_{l}]={\alpha }_{0}+{\sum }_{l=0}^{L}{\beta }_{l}{X}_{l}$, where ${\beta }_{l}$, the coefficient of $l$-day lagged effect, is constrained due to collinearity. We followed an approach proposed by Zanobetti et al. to use a non-parametric function to constrain ${\beta }_{l}$ [27]. In this model, estimated ${\beta }_{0}$ provides the association between $Y$ and concurrent cases of $X$, and estimated ${\sum }_{l=0}^{q}{\beta }_{l}$ provides the $q$-day cumulative effect on the number of DTC orders. That is, the overall impact of a case increase over the next $q$ days is given by ${\sum }_{l=0}^{q}{\beta }_{l}$.

We also examined the presence of weekly seasonality of DTC test orders. Weekly seasonality was modeled using a Poisson regression model on number of DTC orders with time, a quadratic term of time, and $\mathrm{sin}\left(\frac{2\pi t}{T}\right)$ and $\mathrm{cos}\left(\frac{2\pi t}{T}\right)$, T = 7 days, t = 1 for Monday, t = 2 for Tuesday, etc., for weekly seasonality. All analyses were conducted in R 4.2.1 [28]. 

## Results

### Geospatial distribution of DTC orders

**Fig. 1:**
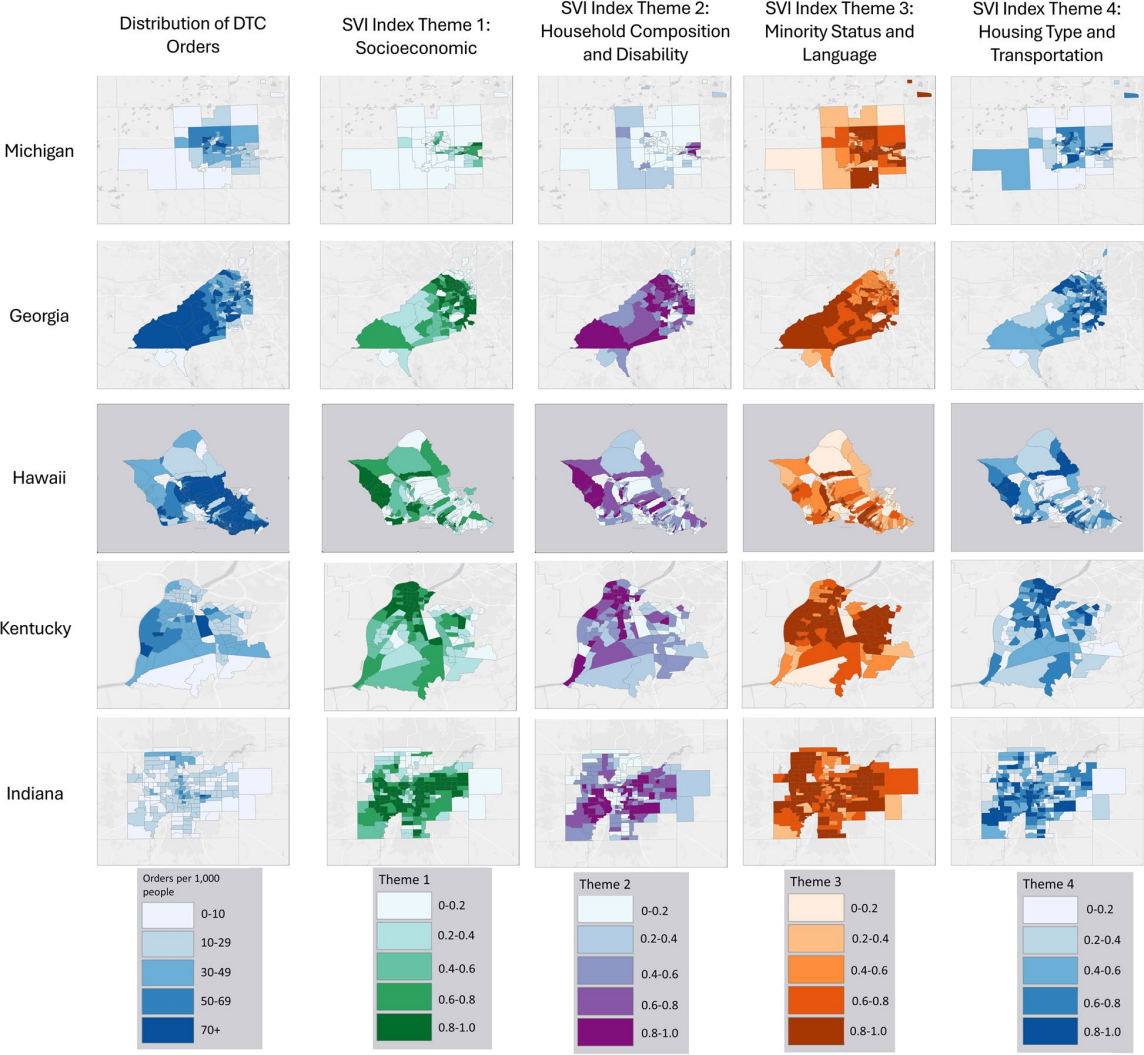
Direct-to-Consumer Order and Sociodemographic Distributions for Intervention Sites Geospatial distribution of direct-to-consumer test kits and Social Vulnerability Themes in intervention communities. Shading represents quintiles of the variables, with dark colors representing the highest quintile and white representing the lowest quintiles

In total, 164,402 households ordered test kits through the digital assistant. DTC orders at all five sites were significantly spatially correlated (Moran’s I: *p* < 0.001) (Fig. 1). After adjusting for block group and census tract-level sociodemographic variables, spatial correlation of DTC orders remained high in Michigan (β = 0.79), Georgia (β = 0.73), Kentucky (β = 0.44), and Indianapolis (β = 0.79). Correlation estimates decreased after adjustment for demographic and socioeconomic variables in Hawaii, though DTC orders remained significantly spatially correlated (unadjusted β = 0.49; adjusted β = 0.19) (Table 1). Sociodemographic factors were variably associated with DTC orders. In Ann Arbor/Ypsilanti, MI, median income and minority status social vulnerability were positively associated with test distribution, indicating that test distribution increased as block-group median income and minority status social vulnerability increased. In Georgia, test distribution increased as block-group population density decreased. Block-group percentage of Bachelor’s degrees was positively associated with test distribution in Georgia, Hawaii, and Indiana; however, it was negatively associated with test distribution in Kentucky. Further, as block-group median age increased, test distribution increased in Georgia, Hawaii, Kentucky, and Indiana. In Hawaii, a substantial amount of clustering was associated to sociodemographic patterns, and test distribution was also positively associated with income and percent of Asian residents. Additionally, as socioeconomic and minority status social vulnerability increased, test distribution decreased in Hawaii. In Kentucky, test distribution was positively associated with population density, and test distribution decreased as housing and transportation social vulnerability increased. Lastly, in Indiana, test distribution was associated with decreasing median income and higher socioeconomic social vulnerability.

**Table 1:** Distribution dates and direct-to-consumer (dtc) orders by intervention community

Community	Distribution Start Date:	Distribution End Date:	Total Kits Distributed	Digital Direct-to-Consumer Test Kit Orders, N (%)	Maximum correlation between DTC orders and Average SARS-CoV-2 Incidence over past 7 days
Ann Arbor/ Ypsilanti, Michigan	June 4, 2021	August 11, 2021	20,000	10,115 (50.6)	0.89
O’ahu, Hawaii	September 19, 2021	September 29, 2021	125,000	79,536 (63.6)	–^a^
Fulton County, Georgia	September 20, 2021	November 1, 2021	51,000	32,537 (63.8)	0.85
Louisville, Kentucky	October 11, 2021	November 13, 2021	40,500	19,204 (47.4)	0.75
Indianapolis, Indiana	October 18, 2021	November 20, 2021	35,300	22,970 (65.1)	-0.01
Total:			271,800	164,402 (60.5)	

^a^Hawaii was excluded from temporal analyses due to the rapid distribution of tests, precluding analyses of distribution over time

### Temporal analysis of test orders

**Fig. 2:**
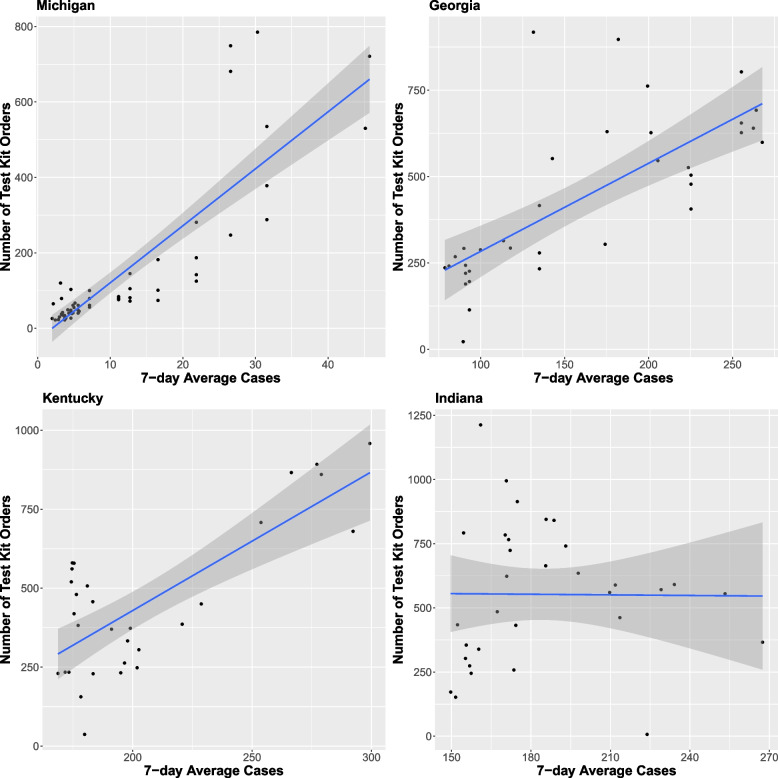
Correlation between Direct-to-Consumer Orders and 7-Day Average SARS-CoV-2 Cases Black dots represent SARS-CoV-2 cases and test kits distributed on each day of the test distribution period. Blue line models the correlation between daily tests distributed and SARS-CoV-2 cases. Grey shaded band represents 95% confidence interval. DTC: direct-to-consumer

DTC orders were variably associated with community incidence of COVID-19 (eFigure 1). We observed strong linear correlations (r > 0.75, *p*-value < 0.001) between DTC orders and same-day COVID-19 incidence in Kentucky, Georgia, and Michigan, with DTC orders increasing with increased community Covid-19 incidence (Table 2, Fig. 2). There was no linear correlation between same-day DTC orders and COVID-19 incidence in Indiana (r = -0.01, *p*-value = 0.96). When we adjusted for previous COVID-19 incidence in the past two weeks, the DTC orders were positively linearly associated with same-day COVID-19 incidence, even in Indiana (effect size = 6.42, 95% CI = 1.32 to 11.52). Distributed lag modeling also indicated a positive 6-day cumulative linear association in Michigan, Georgia, and Kentucky, such that the incidence of COVID-19 during the current day and previous 6-days would increase current DTC orders by 9.0 (95% CI = 1.7, 16.3), 3.0 (95% CI = 1.3, 4.6), and 6.8 (95% CI = 3.4, 10.2) in Michigan, Georgia, and Kentucky, respectively, while an increase of COVID-19 during the current day and previous 6-days rarely increased current DTC orders (1.1 with 95% CI = -4.1, 6.4) in Indiana (eFigure 2, eFigure 3). Also, in Kentucky, the strongest linear correlation occurred with no lag time.

**Table 2:** Associations between Test Kit Distribution and Sociodemographic Characteristics

		**Ann Arbor/Ypsilanti, MI**	**Fulton, GA**	**Oahu, HI**	**Louisville, KY**	**Indianapolis, IN**
**Geospatial Analysis Estimates**^**a**^						
	Unadjusted Spatial correlation	0.85***	0.79***	0.49***	0.46***	0.83***
	Adjusted Spatial correlation^b^	0.79***	0.73***	0.19***	0.44***	0.79***
American Community Survey (block-group level)	% Bachelor’s degree	0.08	0.40**	2.08***	-0.79***	0.51***
	Median Income	0.22***	-0.05	0.46***	-0.07	-0.14**
	Median Age	0.00	0.04*	0.11*	0.03*	0.05***
	Population Density	0.02	-0.06**	-0.02	0.03*	0.01
	% Black	0.18	0.12	-0.07	0.84***	0.10
	% White	0.20	-0.09	0.41	0.97***	0.18
	% Asian	-0.02	-0.17	0.62**	0.80**	-0.16
Social Vulnerability Index Themes (census tract level)	Socioeconomic	0.16	-0.11	-0.03***	0.63	1.38**
	Household Composition and Disability	-0.47	0.23	0.10	0.19	-0.03
	Minority Status and Language	1.13**	0.00	-0.03***	0.36	-0.65
	Housing type and transportation	0.00	-0.12	-0.07	-1.29***	-0.05

^*^p < .05; **p < 0.01; ***p < 0.001

^a^results based on spatially adjusted regression using Queen weighting

^b^adjusted for percent of adults with a bachelor’s degree, Median income, median age, population density, percent of the population that is Black, White, and Asian at the block group level, and CDC’s Social Vulnerability Index themes 1–4 (Socioeconomic status, household composition and disability, minority status and language, and housing type and transportation) at the census tract level

*SVI* Social Vulnerability Index

**Fig. 3:**
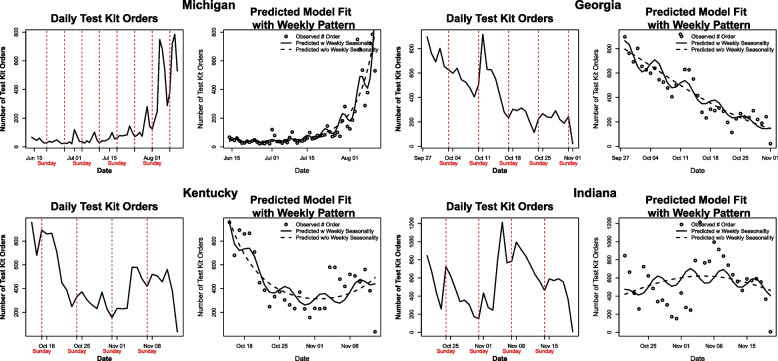
Weekly Seasonality of Direct-to-Consumer Orders Left images portray direct-to-consumer test kit orders by day. Right images portray modeled results. Black line represents predicted direct-toconsumer orders assuming weekly seasonality. Dashed black line indicates predicted direct-to-consumer orders assuming no weekly seasonality. DTC: direct-to-consumer

The weekly seasonality factor was statistically significant (p < 0.001) in all sites. Except Michigan, DTC orders peaked shortly after the weekend, with the highest orders occurring on Mondays and Tuesdays (Fig. 3). In Michigan, however, orders peaked shortly before the weekend, on Thursdays and Fridays.

## Discussion

This is the first study to document the DTC distribution and geospatial correlations of Ag-RDT kits. This study revealed three important findings: 1) DTC orders were spatially clustered in all communities; however, with the exception of Hawaii, selected sociodemographic factors did not explain the clustering observed; 2) DTC orders were associated with community incidence of COVID-19; and 3) Except for Michigan, DTC orders were more common shortly after the weekend, on Monday and Tuesdays. These findings may inform future strategies for Ag-RDT distribution.

In terms of spatial clustering, sociodemographic factors contributed substantially in Hawaii, but not so elsewhere. The remaining clustering may be explained by different DTC implementation, marketing approaches, and internet access, as well as differing local health department involvement [15]. Each community was given autonomy to share information about the program in whatever approach they preferred (i.e., Facebook posts, television, radio advertisements, or community testing events). Additionally, other community-level demographic factors, including age, race, and educational attainment, were inconsistently associated with DTC orders, with no clear trends across sites. While higher levels of social vulnerability were associated with increased test distribution in Michigan and Indiana, test distribution was decreased within socially vulnerable areas in Kentucky and Hawaii. This is an intriguing finding because it suggests no consistent, clear gaps in DTC orders among Black populations, rural populations, or those with low socioeconomic status, populations that have previously been reported to have decreased access to COVID-19 testing [3, 4, 7, 29]. However, these findings should be interpreted with caution, as we limited our analysis to census track and not self-reported sociodemographic factors of the people who placed DTC orders, which could bias the results. It is also important to note that this study only reports Ag-RDT kit *orders*, and it is unknown how recipients used their tests. A study of over 400,000 individuals throughout the United States from August 2021 to March 2022 found that White individuals were nearly twice as likely to use Ag-RDT, and use of Ag-RDT increased with increasing income and education [6]. Therefore, while DTC orders may increase access to Ag-RDT, to understand how Ag-RDT testing behaviors and attitudes may vary across sociodemographic groups, resulting in disparities in test completions.

This study also highlights the relationship between DTC orders and the community incidence of COVID-19. We most clearly observed this pattern in Michigan, where the intervention took place during the surge of the Delta variant in the summer of 2021. As the community incidence of COVID-19 increased throughout the intervention period, DTC test kit orders also increased. The observed lag between increasing community incidence and increase in DTC orders, ranging from 6–10 days in Michigan and Georgia, likely represents consequent transmission, symptom recognition, and accompanying increase in testing demand. Further, the weekly seasonality of orders indicates that people often frame their orders around the weekend, a time of socialization and COVID-19 exposure. Future testing interventions should use this information to account for increased demand in the days during or immediately following the weekend.

In the United States, Ag-RDTs for COVID-19 are an important part of the Federal Government’s approach to manage the ongoing COVID-19 pandemic. The U.S. government aimed to distribute over 1 billion free Ag-RDTs across the United States in 2022, using similar online DTC distribution methods [30]. As the distribution of Ag-RDTs is scaled up, it is important to maintain a focus on equity, to ensure test distribution is accessible and available to those who need them most. This requires ongoing monitoring of testing, including access, completion, and reporting activities, at the neighborhood level.

### Strengths and limitations

This study offers a unique look at socio-demographics of DTC ordering in a large, community-wide COVID-19 testing program across multiple U.S states. Currently, very little is known about the ordering of Ag-RDTs, and this knowledge is critical for the design of future public health interventions and programs. However, there are limitations in this study. First, some individual and community level variables of interest, including internet access, phone/computer behavior, and census block-group-level COVID-19 vaccination rates, were not available for inclusion in analyses. Second, we are assuming that the population who ordered DTC tests reflects the Census demographics of the district; however, we do not have demographic data for individual test users. This data should be interpreted with this in mind to avoid ecological fallacy. This may contribute to error with our ability to detect demographic patterns with DTC orders. Demographic disparities in test completion could not be evaluated, though this could contribute to disparities in COVID-19 outcomes. Lastly, while the five communities included represent a geographically diverse collection of U.S. communities, the nationwide generalizability of these findings may be limited, and additional research is necessary to understand rapid antigen distribution patterns throughout the country.

## Conclusion

As Ag-RDTs become increasingly widespread, it is important to understand ordering and uptake of Ag-RDT to ensure equitable access to testing. We did not find sociodemographic characteristics related to DTC ordering behavior. Test ordering was geospatially correlated based on census track data and largely irrespective of sociodemographic characteristics, suggesting that digital distribution alongside community engagement may be an effective strategy for equitable large-scale distribution.

### Supplementary Information

**Additional file 1:** **eFigure 1.** Direct-to-Consumer Orders and 7-day Average COVID-19 Incidence throughout the Distribution Period. **eFigure 2.** Estimated Lag Effects between Number of Direct-to-Consumer Orders and t-day Lagged COVID-19 Cases. 3. **eFigure 3.** Normalized Estimates from Distributed Lag Model.

## Data Availability

All data and code used in this study are available from study authors upon reasonable request to Apurv.soni@umassmed.edu.

## Abbreviations

DTC: Direct-to-consumer
Ag-RDT: Rapid antigen diagnostic tests
SYCT: Say Yes! Covid Test
