# Supplementary material for: Association of neighborhood-level sociodemographic factors with Direct-to-Consumer (DTC) distribution of COVID-19 rapid antigen tests in 5 US communities
Source: BMC Public Health. 2023 Sep 22;23:1848. doi: 10.1186/s12889-023-16642-3 (PMC10515232; doi:10.1186/s12889-023-16642-3)
Supplement: Supplementary file 1 — Additional file 1: eFigure 1. Direct-to-Consumer Orders and 7-day Average COVID-19 Incidence throughout the Distribution Period. eFigure 2. Estimated Lag Effects between Number of Direct-to-Consumer Orders and t-day Lagged COVID-19 Cases. 3. eFigure 3. Normalized Estimates from Distributed Lag Model. [file 12889_2023_16642_MOESM1_ESM.docx]

Online Supplemental Figures

[**eFigure 1**: Direct-to-Consumer Orders and 7-day Average COVID-19 Incidence throughout the Distribution Period 2](#_Toc129689336)

[**eFigure 2**: Estimated Lag Effects between Number of Direct-to-Consumer Orders and t-day Lagged COVID-19 Cases 3](#_Toc129689337)

[**eFigure 3**: Normalized Estimates from Distributed Lag Model 4](#_Toc129689338)

## **eFigure 1**: Direct-to-Consumer Orders and 7-day Average COVID-19 Incidence throughout the Distribution Period, 2021


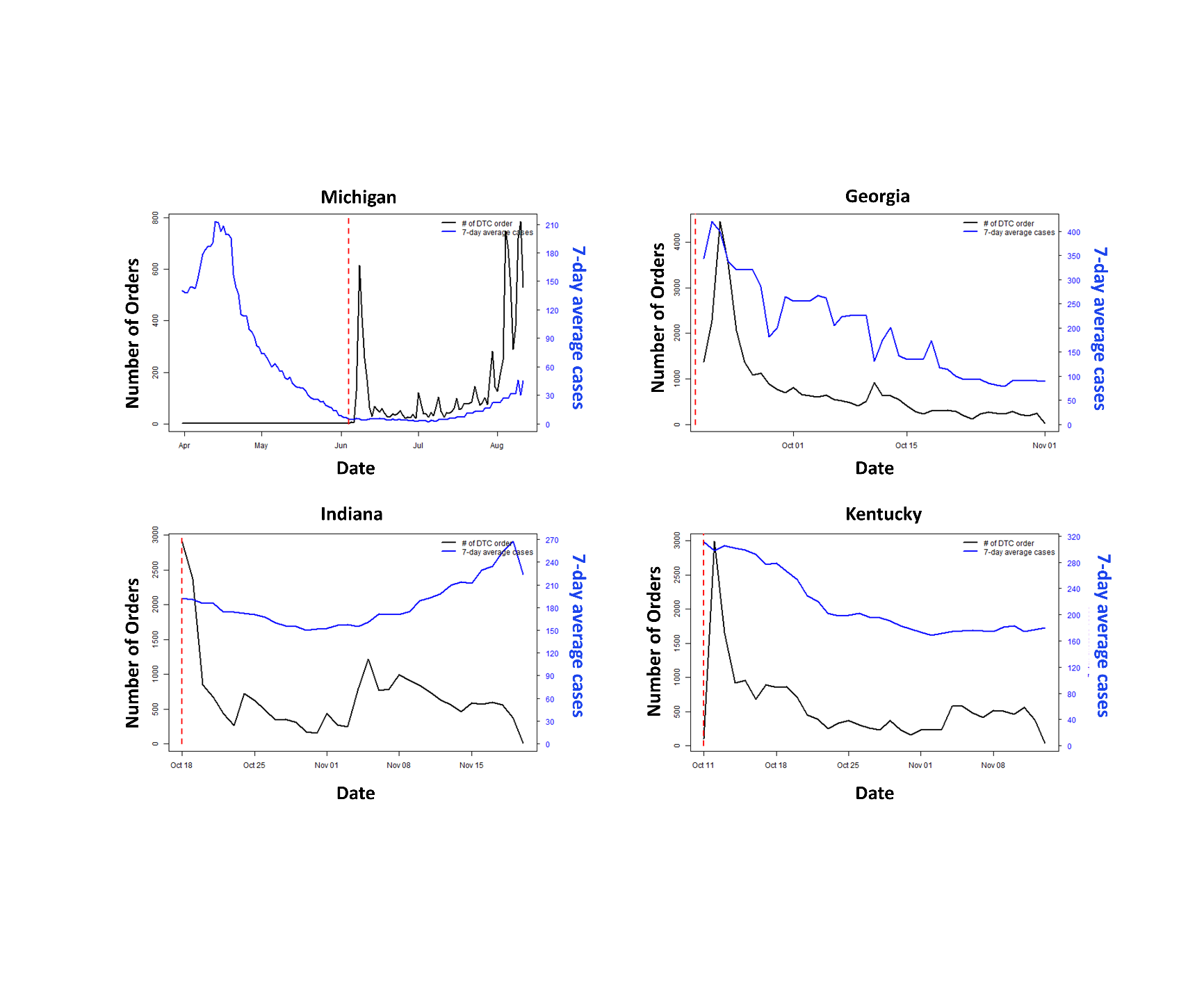


Vertical red dotted line represents first day of test kit distribution. Blue line and left y-axis show 7-day average incidence of SARS-CoV-2 at the state level as reported through the Johns Hopkins COVID-19 Tracker.^1^ Black line shows number of direct-to-consumer (DTC) test kit orders.

## **eFigure 2**: Estimated Lag Effects between Number of Direct-to-Consumer Orders and t-day Lagged COVID-19 Cases


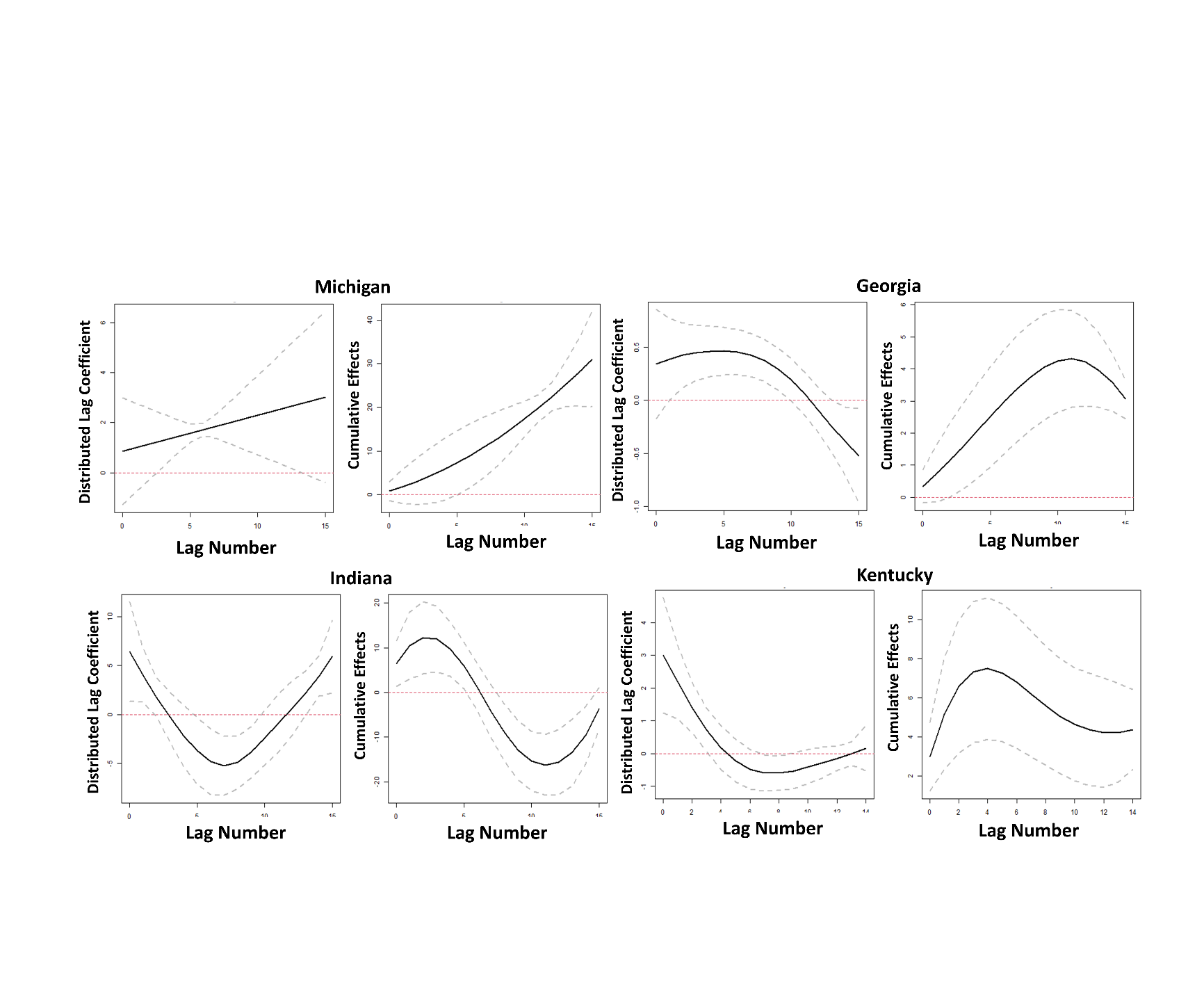


Dashed lines represent 95% confidence intervals. Left panels show coefficients from distributed lag model. Right panels show cumulative effects over lag period.

## **eFigure 3**: Normalized Estimates from Distributed Lag Model


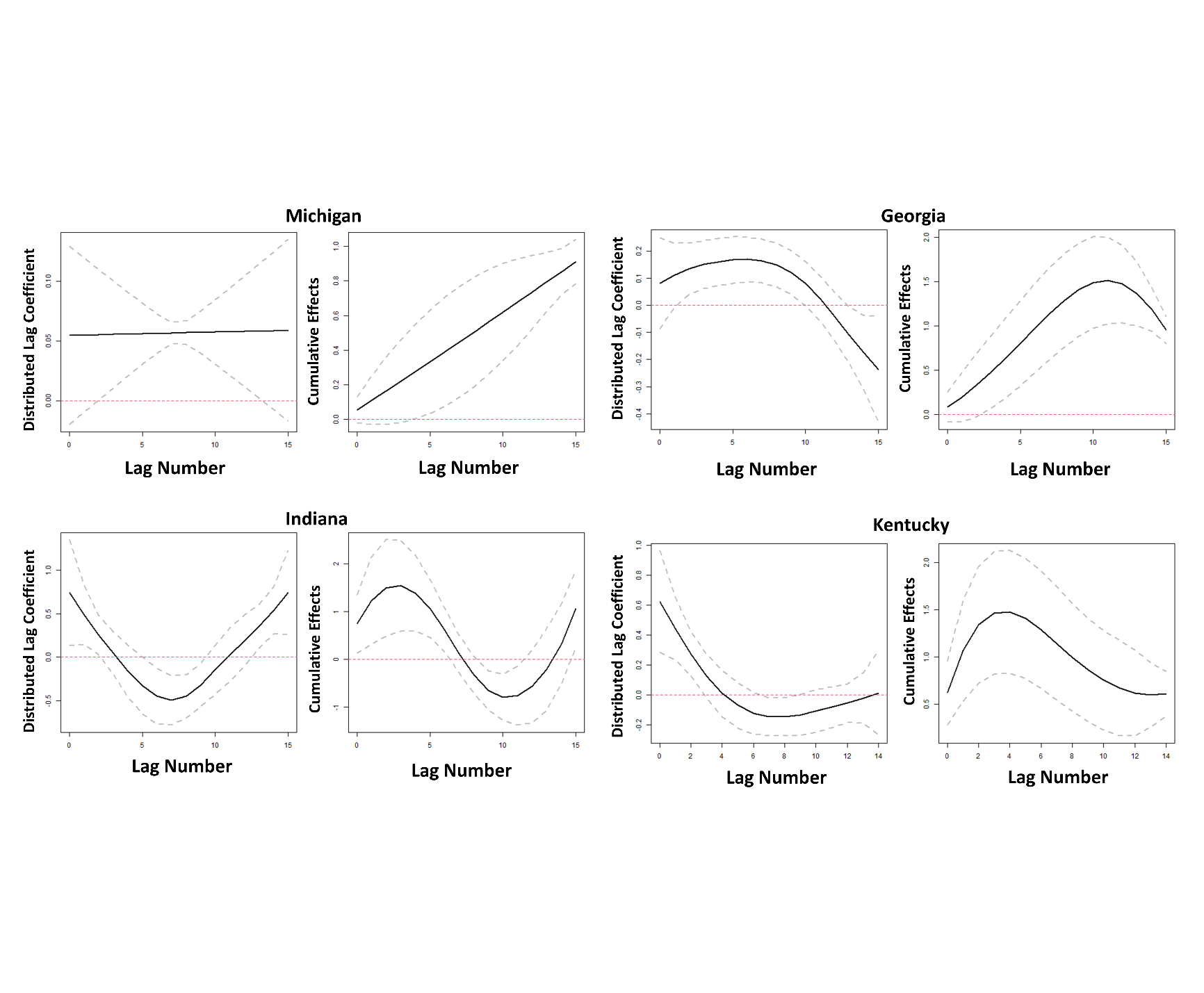


Dashed lines represent 95% confidence intervals. Left panels show coefficients from distributed lag model. Right panels show cumulative effects over lag period.
